# Impact of physical activity and exercise on bone health in patients with chronic kidney disease: a systematic review of observational and experimental studies

**DOI:** 10.1186/s12882-020-01999-z

**Published:** 2020-08-08

**Authors:** Daniela F. Cardoso, Elisa A. Marques, Diogo V. Leal, Aníbal Ferreira, Luke A. Baker, Alice C. Smith, João L. Viana

**Affiliations:** 1 Research Center in Sports Sciences, Health Sciences and Human Development, CIDESD, University Institute of Maia, Av. Carlos Oliveira Campos - Castelo da Maia, 4475-690 Maia, Portugal; 2 Department of Nephrology, Curry Cabral Hospital, Lisbon, Portugal; 3 Leicester Kidney Lifestyle Team, Department of Health Sciences, University of Leicester, Leicester, UK

**Keywords:** Physical activity, Exercise, Bone, CKD-MBD

## Abstract

**Background:**

Chronic Kidney Disease (CKD) patients frequently develop life-impairing bone mineral disorders. Despite the reported impact of exercise on bone health, systematic reviews of the evidence are lacking. This review examines the association of both physical activity (PA) and the effects of different exercise interventions with bone outcomes in CKD.

**Methods:**

English-language publications in EBSCO, Web of Science and Scopus were searched up to May 2019, from which observational and experimental studies examining the relation between PA and the effect of regular exercise on bone-imaging or -outcomes in CKD stage 3–5 adults were included. All data were extracted and recorded using a spreadsheet by two review authors. The evidence quality was rated using the Cochrane risk of bias tool and a modified Newcastle-Ottawa scale.

**Results:**

Six observational (4 cross-sectional, 2 longitudinal) and seven experimental (2 aerobic-, 5 resistance-exercise trials) studies were included, with an overall sample size of 367 and 215 patients, respectively. Judged risk of bias was low and unclear in most observational and experimental studies, respectively. PA was positively associated with bone mineral density at lumbar spine, femoral neck and total body, but not with bone biomarkers. Resistance exercise seems to improve bone mass at femoral neck and proximal femur, with improved bone formation and inhibited bone resorption observed, despite the inconsistency of results amongst different studies.

**Conclusions:**

There is partial evidence supporting (i) a positive relation of PA and bone outcomes, and (ii) positive effects of resistance exercise on bone health in CKD. Prospective population studies and long-term RCT trials exploring different exercise modalities measuring bone-related parameters as endpoint are currently lacking.

## Background

Chronic kidney disease (CKD) is a worldwide health problem with an estimated global prevalence of 11–13% [1]. This prevalence is rising, driven by an aging population and the increasing incidence of obesity, hypertension and diabetes [1]. In addition, most patients have an increased risk of comorbidities [2] and all-cause cardiovascular premature death [3]. As a result, CKD represents an enormous economic burden for healthcare systems worldwide with drastic personal health consequences [1]. Patients suffering from CKD frequently develop mineral and bone disorders (MBD) due to systemic alterations induced by the disease [4]. This syndrome has been associated with the spectrum of renal osteodystrophy [4], vascular calcifications, abnormalities in bone mineralisation and turnover [5], increased bone fractures [4], as well as increased morbidity and mortality, resulting in a diminished quality of life [6]. Thus, CKD-MBD encompasses a wide spectrum of clinical disorders such as alterations in mineral and bone metabolism [6], which are in turn associated with abnormalities in calcium, parathyroid hormone (PTH), phosphate or vitamin D metabolism [7]. For instance, reduced levels of vitamin D and osteocalcin (OC) carboxylation, and elevated serum PTH and fibroblast growth factor 23 (FGF-23) are key risk factors for bone disease [8]. PTH and FGF-23 are the main regulating hormones of bone integrity and mineral homeostasis [2].

Bone is a dynamic tissue which is constantly undergoing remodelling [9], a process that mediates the balance between bone formation and resorption to maintain bone health and skeleton integrity [10]. However, in CKD-MBD the rate of bone resorption exceeds the rate of bone formation, resulting in loss of bone quantity and quality, which consequently contributes to bone strength loss [10].

Different physical activities, including high-impact weight-bearing exercise, multi-directional weight-bearing exercise, or resistance exercise have been pointed as potentially able to stimulate resident osteocytes to yield signalling molecules that regulate bone formation and bone resorption [11, 12]. In addition, substantial evidence supports that physical activity (PA) and exercise interventions are effective in improving bone health across all ages [13, 14]. Although different exercise interventions (varying on type, intensity, frequency, and duration) have been extensively explored in healthy and osteoporotic populations [13, 15], the impact of PA and exercise on bone health in CKD patients is less well-established. As there has been no definitive synthesis of these studies, the current systematic review makes a major contribution to research through the inclusion of observational and experimental studies, in order to explore the impact of different forms of mechanical loading on different imaging and biochemical bone outcomes in CKD. Thus, the purpose of the present systematic review is to examine (1) the associations between PA and bone-related outcomes and (2) the effects of different exercise interventions on bone-related outcomes in CKD patients.

## Methods

### Eligibility criteria

The inclusion criteria for this systematic review were: (1) observational studies, or randomized controlled trials (RCTs), or non-randomized controlled trials (non-RCTs); (2) reported measures of PA or implemented an exercise intervention as the only intervention; (3) reported data on one or more of the following bone outcomes: bone density, geometry, microarchitecture, and biomarkers of bone turnover; (4) adult CKD patients (age ≥ 18 years old); and (5) CKD stage 3 to 5, including patients under dialysis or kidney transplant recipients.

We did not include review articles, editorials, conference abstracts or animal-based trials. Studies published in non-English-language were also not included due to potential errors in the translation and interpretation of findings.

Bone parameters were defined as areal bone mineral density (BMD) or bone mineral content (BMC) or T-score measured with dual X-ray absorptiometry (DXA), bone macro- and micro-structure measured by 3D imaging techniques [quantitative computerized tomography (QCT) and magnetic resonance imaging (MRI)], and quantitative ultrasound (QUS) measurements of bone density that included broadband ultrasound attenuation (BUA) and the speed of sound (SOS). All skeletal sites were considered. Bone outcomes included any formation, resorption and regulators markers of bone turnover measured using any detection technique.

Bone outcomes based on conventional radiography and bone biopsies were not included.

### Search strategy and data source for studies identification

This systematic review is in accordance with the Preferred Reporting Items for Systematic Reviews and Meta-Analyses: The PRISMA Statement guidelines (Additional file 1) [16]. A computer search of databases was conducted on EBSCO, Web of Science and Scopus up to May 2019. The search terms used were: “CKD”, “dialysis”, “renal function”, “glomerular filtration rate”, “hemodialysis”, “renal”, “bone”, “exercise”, “physical activity” (supplementary search strategy in Additional file 2). At this stage, there were no limits on the search, such as, language, animal and human-based study, or age. Hand searching on Google Scholar was also performed to identify possible missed studies in database search. The reference lists of all the included studies have also been examined to identify any potential missed studies. Afterwards, all the duplicate data were identified and removed through the use of a reference management software (EndNote®, version X7.8).

### Data extraction

Data extraction was completed using a spreadsheet to record information on a range of characteristics of each study, including: first author and publication year, country, study design, sample size, type of population (stage of CKD), outcomes measured, description of PA assessment, description of the exercise intervention (for RCTs and non-RCTs) and main results for each outcome. Data were independently extracted by two review authors (DC and EAM) and in case of missing or unclear information, the authors of the included studies were contacted for further details.

### Methodological quality

The risk of bias of the included studies was assessed using an adapted version of a modified Newcastle-Ottawa Scale (NOS) tool for observational studies [17] and the Cochrane Collaboration tool for the experimental studies [18]. The NOS includes the following five domains: methods for the selection of participants (selection bias), methods to control for confounding (performance bias), statistical methods (detection bias), methods of measuring outcome variables (information bias), and subject follow-up (attrition bias for longitudinal studies). Instead of using the scale 0 (for high risk of bias), 1 (for mostly no), 2 (for mostly yes) and 3 (for low risk of bias) as previously described [19], judgements were categorized as ‘low risk’ of bias, ‘moderate risk’ of bias, ‘high risk’ of bias or ‘unclear or unknown risk’ of bias following our adapted version (Additional file 3). The Cochrane risk of bias tool [18] addresses the following six domains: selection bias (random sequence generation and allocation concealment), performance bias (blinding of participants and personnel), detection bias (blinding of outcome assessment), attrition bias (incomplete outcome data), reporting bias (selective reporting) and other bias. For each entry, judgements were categorized as ‘low risk’ of bias, ‘high risk’ of bias, or ‘unclear risk’ of bias.

Two authors (DC and EAM) independently scored each of the included articles and discrepancies were resolved through discussion until consensus was met.

## Results

### Included studies

**Fig. 1:**
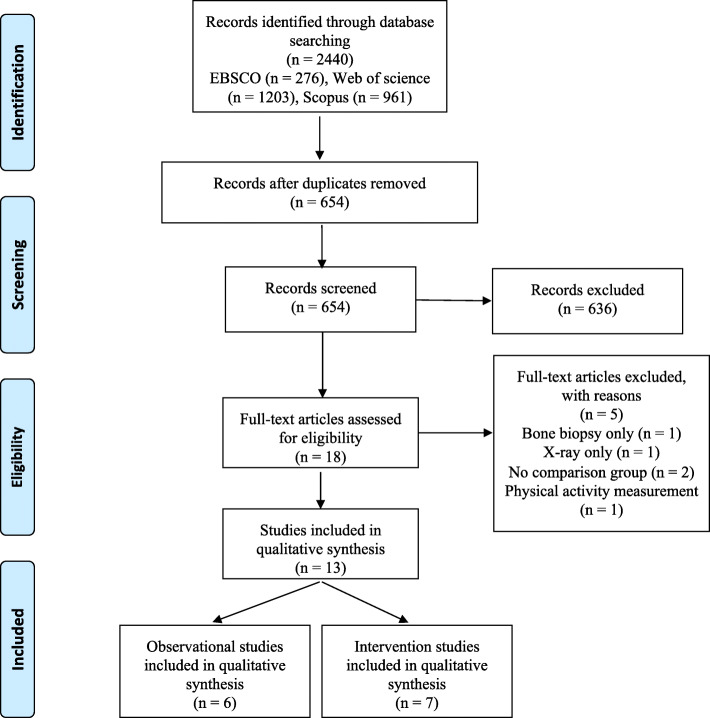
Flow diagram of studies

Figure 1 shows the flowchart of the search and selection process. A total of 2440 articles were identified by the search strategy. After removing duplicate records, the titles, keywords and abstracts of 654 articles were analyzed and 18 relevant articles were identified for full text review. From those, 13 studies fulfilled the inclusion criteria and were included in our qualitative synthesis. Studies were classified based on inherent purpose and design features in observational (*n* = 6) or experimental (*n* = 7).

### Characteristics of the observational studies

The age range of our analytical sample in the observational studies was between 19 and 85 years old, mostly representing middle-aged adults and older adults. The characteristics of the included six observational studies [20–25] are presented in Table 1. Four studies had a cross-sectional design [20, 22, 24, 25], and two had a longitudinal design [21, 23] with an observational period of 12 and 24 months, respectively. Only one study had a multi-centre design [23]. Regarding patients characteristics, four reports recruited patients under haemodialysis (HD) treatment [22–25] and two studies were performed on kidney transplant patients [20, 21]. Sample size from individual studies ranged from 32 to 115 patients, and the overall sample size was 367 participants. Median age of participants was 56 years (based on the reported mean age), which varied from 19 to 85 years.

**Table 1:** Characteristics of the observational studies

Study (design)	Country	Sample size (male %); Population	BMD assessment techniques (anatomical sites)	Bone biomarkers	PA assessment method	Results BMD	Results Bone biomarkers
Dolgos et al. 2008 [20] (Cross-sectional)	Norway	*n* = 108 (68%) Kidney Transplant	DXA – Lunar (LS, proximal femur both sides, and total body)	iPTH	Self-report questionnaire Physical active vs. physical Inactive (defined as regular weight-bearing physical exercise performed at least twice a week for 30 min)	Association with total body BMD No association with LS and proximal femur BMD	No association with iPTH
Huang et al. 2009 [22] (Cross-sectional)	Taiwan	*n* = 35 HD	DXA – Hologic (LS and FN)	iPTH ALP	Self-report interview questionnaire Total weekly exercise time (min/week): regular exercise (yes or no), exercise type (impact or non-impact) and effective exercise time (min/week)	Positive association with all BMD outcomes	No association with all bone biomarkers
Morishita et al. 2014 [24] (Cross-sectional)	Japan	*n* = 32 (56%) HD	–	BALP iP1NP TRAP-5b iPTH	Device- Triaxial pedometer Vigorous and moderate PA volumes per week	–	No association with all bone biomarkers
Ota et al. 1997 [25] (Cross-sectional)	Japan	n = 32 (0%) HD	DXA – Lunar (Total body and LS)	iPTH OC ALP TRAP-5b	Device – Accelerometer Total energy expenditure per day (Kcal) for 7 days - mean energy expenditure per day	Positive association with total body BMD No association with LS BMD	No association with all bone biomarkers
Groth et al. 1995 [21] (Longitudinal - 2-year follow-up)	Germany	*n* = 115 (61%) Kidney Transplant	DXA- Lunar (LS)	ALP iPTH OC	Self-report questionnaire Estimated energy spending during sports	Positive association with bone gain (r = 0.2, *p* < 0.05)	No association with all bone biomarkers
Malluche et al. 2017 [23] (Longitudinal - 1-year follow-up)	USA	*n* = 45 HD	DXA - Lunar and QCT (LS and proximal femur)	TRAP-5b; BALP P1NP; Sclerostin DKK1; FGF-23 iPTH	Self-report questionnaire Exercised 1+ days/week	No association with bone loss	No association with all bone biomarkers

*ALP* Alkaline Phosphatase, *BALP* Bone-specific Alkaline Phosphatase, *BMD* Bone Mineral Density, *DKK1* Dickkopf-related protein 1, *DXA* Dual-energy X-ray absorptiometry, *FGF-23* Fibroblast Growth Factor 23, *FN* Femoral Neck, *HD* Haemodialysis, *iPTH* Intact Parathyroid Hormone, *LS* Lumbar Spine, *OC* Osteocalcin, *PA* Physical Activity, *P1NP* Procollagen type I N-terminal Propeptide, *TRAP-5b* Tartrate-resistant acid phosphatase 5b

The most common method to assess PA was the use of self-report questionnaires [20–23], while objective measures were only captured in two studies [24, 25], using triaxial pedometry and triaxial accelerometry, respectively.

Except for one cross-sectional study in HD patients [24], areal BMD (g/cm^2^) was measured trough DXA in all included observational studies. Only one study also used QCT, a 3D imaging technique, to assess total volumetric BMD (g/cm^3^) at the proximal femur and spine, and cortical or trabecular mass (g) and volume (cm^3^) at the proximal femur [23]. Most studies measured areal BMD at more than one skeletal site. Lumbar spine BMD was assessed in all studies, while proximal femur was assessed in two of these studies [20, 23], total body BMD was measured in two studies [20, 25] and one study also measured femoral neck BMD [22]. In addition to imaging-derived bone parameters, different biochemical markers of bone metabolism were measured in all observational studies included in this review. Bone formation markers included alkaline phosphatase (ALP) [21, 22, 25], bone-specific alkaline phosphatase (BALP) [23, 24], OC [21, 25] and procollagen type I N-terminal propeptide (P1NP) [23] and intact P1NP (iP1NP) [24]. Whereas, one or more studies included data on bone resorption markers such as sclerostin [23], dickkopf-related protein 1 (DKK1) [23] and tartrate-resistant acid phosphatase isoform 5b (Trap-5b) [23–25]. In addition, FGF-23, a local factor in bone remodelling that stimulates bone formation and resorption, was also assessed in one study [23]. Finally, intact-PTH (iPTH) was reported in all studies.

### Characteristics of the experimental studies

The age range of our analytical sample in the experimental studies was between 27 and 76 years old, mostly representing middle-aged adults and older adults.

We identified five RCTs [26–30] and two non-RCTs [31, 32] aiming to examine the effects of exercise on bone parameters, which are described in detail in Table 2. The sample size of the individual studies varied, ranging between 13 and 52 subjects, and the overall sample size was 215 patients. The median value of the mean age was 52 years and ranged between 27 to 76 years old. Participants were mostly HD patients; only one study included CKD stage 3–4 patients [27], and another study included subjects with history of kidney transplantation [26]. All outcomes were measured at baseline and at the end of each intervention period, corresponding to 8 weeks [29], 12 weeks [26, 28, 30], and 24 weeks [27, 31, 32]. All exercise sessions were supervised by a certified professional, except the home-based exercise trial that was weekly supervised [27]. The most common type of exercise training was resistance exercise, usually performed during dialysis (intradialytic exercise) three times per week [29–32]. Only one study performed resistance exercise for kidney transplant patients [26]. In addition, two studies performed aerobic exercise interventions, three times per week [27, 28], and in one study [28] the aerobic exercise was performed during dialysis.

**Table 2:** Characteristics of the experimental studies

Study (design)	Country	Sample size (male %) Population	BMD assessment techniques (anatomical sites)	Bone biomarkers	Exercise intervention	Results BMD	Results Bone biomarkers
Eatemadololama et al. 2017 [26] (RCT)	Iran	*n* = 24 CG = 12 EG = 12 Kidney Transplant	DXA – Hologic (proximal femur and LS)	–	Resistance exercise (10 min stretching exercises, 10 min walking, 10 min cycling, 20 min RE for UL, 20 min RE for LL; RE intensity 50% of 1RM increasing 5 to 10%; 2 days/week; 12 weeks)	*EG*: ↑ proximal femur = LS *CG*: ↓ proximal femur ↓ LS *EG* vs *CG*: no comparison	–
Gomes et al. 2017 [27] (RCT)	Brazil	*n* = 39 (71%) CG = 15 EG = 24 CKD Stages 3–4	–	TRAP-5b PTH Sclerostin ALP OC	Aerobic exercise (40–60% of maximum VO_2_; 30 min; 3 days/week; 24 weeks)	–	*EG*: ↑ ALP *CG*: = all biomarkers *EG* vs *CG*: ↑ ALP (favouring EG)
Liao et al. 2016 [28] (RCT)	Taiwan	*n* = 40 (43%) CG = 20 EG = 20 HD	DXA (LS and FN)	iPTH	Intradialytic aerobic exercise (12–15 BPES; 30 min; 3 days/week; 12 weeks)	*EG*: = all bone sites *CG*: = all bone sites *EG* vs *CG*: ↑ FN BMD loss (favouring CG)	*EG*: = all biomarkers *CG*: = all biomarkers *EG* vs *CG*: no comparison
Marinho et al. 2016 [29] (RCT)	Brazil	*n* = 13 (46%) CG = 7 EG = 6 HD	–	iPTH BALP Sclerostin	Intradialytic resistance exercise (60–70% of 3RM; 4 exercises; 3 days/week; 8 weeks)	–	*EG*: ↑ BALP *CG*: = all biomarkers *EG* vs *CG*: no comparison
Rosa et al. 2018 [30] (RCT)	Brazil	*n* = 52 (67%) CG = 24 EG = 28 HD	DXA – Hologic (Total body - BMC)	–	Intradialytic resistance exercise (60% of 1RM; 40-50 min; 3 days/week; 12 weeks)	*EG*: = total body BMC *CG*: = total body BMC *EG* vs *CG*: ↑ BMC (favouring EG) Effect size = 0.65	–
Marinho et al. 2017 [32] (Non-RCT)	Brazil	*n* = 26 (65%) CG = 12 EG = 14 HD	–	OC OPN OPG iPTH	Intradialytic resistance exercise (60–70% of 1RM; 4 exercises; 3 sets; 10 repetitions; 3 days/week; 24 weeks)	–	*EG*: ↑ OPG *CG*: = all biomarkers *EG* vs *CG*: no comparison
Marinho et al. 2016 [31] (Non-RCT)	Brazil	*n* = 21 (67%) CG = 11 EG = 10 HD	DXA – Lunar (FN, LS, proximal femur and total body)	PTH	Intradialytic resistance exercise (60–70% of 1RM; 4 exercises; 3 sets; 10 repetitions; 3 days/week; 24 weeks)	*EG*: ↑ femoral neck *CG*: = all bone sites *EG* vs *CG*: no comparison	*EG*: = PTH *CG*: no comparison *EG* vs *CG*: no comparison

*ALP* Alkaline Phosphatase, *BALP* Bone-specific Alkaline Phosphatase, *BMD* Bone Mineral Density, *BMC* Bone Mineral Content, *BPES* Borg Perceived Exertion Scale, *CG* Control Group, *DXA* Dual-energy X-ray absorptiometry, *EG* Exercise Group, *FN* Femoral Neck, *HD* Haemodialysis, *iPTH* Intact Parathyroid Hormone, *LL* Lower Limb, *LS* Lumbar Spine, *OC* Osteocalcin, *OPG* Osteoprotegerin, *OPN* Osteopontin, *P1NP* Procollagen type I N-terminal Propeptide, *PTH* Parathyroid Hormone, *RE* Resistance Exercise, *RM* One Repetition Maximum, *TRAP-5b* Tartrate-resistant acid phosphatase 5b, *UL* Upper Limb, ↑ significant increase, = no change, ↓ significant decrease

Except for two experimental studies with no imaging-derived bone parameters [27, 29], all other five studies measured BMD through DXA devices [26, 28, 30–32]. Two studies reported T-score values [31] and BMC [30] from total body scans. All other studies reported DXA-derived outcomes from two or more skeletal sites. Areal BMD was reported for lumbar spine in three studies [26, 28, 31], for proximal femur in two studies [26, 31], and for femoral neck in two studies [28, 31]. One study reported only the T-score values for lumbar spine, proximal femur, femoral neck and total body [31]. In addition to imaging-derived bone parameters, different biochemical markers of bone metabolism were measured in five experimental studies [27–29, 31, 32], including the following markers of bone formation: ALP [27], BALP [29], and OC [27, 32]; while markers of bone resorption included sclerostin [27, 29], Trap-5b [27], and osteoprotegerin (OPG) [32]. Other biomarkers of bone health were also reported, including osteopontin (OPN) [32], iPTH [28, 29, 32] and PTH [27, 31].

### Risk of bias in the observational studies

In total, six observational studies were examined in the present review. From these, overall assessment showed that one study had low [20] and another had moderate [23] risk of bias, with the remaining revealing high risk of bias [21, 22, 24, 25], as seen in Additional file 4. Furthermore, all studies examined bone biomarkers and BMD as the main outcomes, except for Morishita et al., 2014 [24], and the summary assessment for outcome replicates the overall summary assessment for all observational studies.

Examining each domain separately, high risk is attributed to bias in Selection and bias in Performance only. All other domains were rendered as low risk of bias.

### Risk of bias in the experimental studies

This present review has examined seven experimental studies. Determination of the summary of overall risk of bias in each study shows that from these, three studies were judged to have high risk of bias [30–32], whereas the remaining four studies [26–29] are classified as unclear risk of bias (Additional file 5). Additionally, when examining the risk of bias for outcome, risk of bias was unclear [26, 28] and high [30, 31] in the four studies that examined BMD. Bone biomarkers have been examined in five of the seven experimental studies, with overall unclear [27–29] and high [31, 32] risk of bias observed.

On the other hand, when examining each domain individually (i.e. risk of bias across studies), Reporting and Attrition bias were judged as low risk, whereas all other domains may be interpreted as having unclear risk of bias.

### Association between physical activity and bone outcomes (observational studies)

Based on cross-sectional studies results, higher PA was associated with higher DXA-derived areal BMD (g/cm^2^) measured at different skeletal sites. Associations were more consistent for total body [20, 25] and femoral neck [22], but only three studies explored these outcomes. Only one of the three cross-sectional studies that measured lumbar spine BMD in HD patients found a significant association with exercise duration (min/week) [22].

Two longitudinal studies reported the association between PA and bone mass changes, and findings were inconsistent. Only one study explored this association with proximal femur bone loss (areal BMD, cortical and trabecular volume and mass) and found no evidence of association with PA [23]. Similarly, to cross-sectional data, estimates of association between PA and lumbar spine bone loss pointed for inconsistent results. A positive correlation between estimated energy expenditure during sports with bone gain (*r* = 0.2, *P* < 0.05) was reported in kidney transplant patients [21], while a lack of association was reported with two-year spinal BMD loss measured by QCT or DXA [23] in HD patients. Finally, the results for biochemical markers of bone metabolism were consistent, with all cross-sectional and longitudinal studies reporting no significant associations. In summary, cross-sectional data shows positive associations between PA and BMD at femoral neck, lumbar spine and total body. Evidence from longitudinal studies was conflicting, with only one study supporting a positive association between PA and lumbar spine BMD gain. Based on all included observational studies, PA is not related with bone metabolism biomarkers in CKD patients.

### Exercise-related effects on bone outcomes (experimental studies)

Only one study explored the effect of aerobic exercise (intradialytic) on BMD and found no significant differences at both lumbar spine and femoral neck areal BMD in the exercise and control groups after 12 weeks [28]. Of note, this study reported a significant bone loss at the femoral neck in the control group compared to the exercise group.

Data from trials evaluating the effects of resistance exercise protocols reported inconsistent results on areal BMD at different skeletal sites. Taken together, results suggested an increase – particularly at the femoral neck and proximal femur [26, 31] – or no significant changes in areal BMD after resistance exercise training, mostly at lumbar spine and total body [26, 30, 31].

Based on the results of the five studies reporting data on biochemical markers of bone metabolism, only three studies found a significant effect of exercise in a limited number of bone biomarkers linked to bone formation (ALP and BALP) and bone resorption (OPG) [27, 29, 32]. The significant increase in ALP levels were observed only in the exercise groups after 24 weeks of aerobic exercise in CKD stages 3–4 patients [27] and in BALP after 8 weeks of resistance exercise in HD patients [29]. In addition, 24 weeks of intradialytic resistance exercise significantly improved OPG levels, while no changes were observed in the control group [27]. Levels of OC, Trap-5b and OPN were consistently unchanged following any type of exercise intervention [27, 29, 32].

## Discussion

### Summary of the main evidence

The current systematic review showed that PA may be associated with BMD in HD and kidney transplant patients. However, these findings are mostly supported by cross-sectional data showing positive associations between PA and BMD, despite evidence from longitudinal studies was contradictory (i.e. the analysis of observational studies proposes that PA is not related with bone metabolism biomarkers in CKD patients). Evidence from the experimental studies highlights exercise interventions as beneficial to improve bone health in CKD stages 3–4, HD and kidney transplant patients improve bone health in CKD stages 3–4, HD and kidney transplant patients, with resistance exercise training drawing more solid conclusions than aerobic exercise in its potential effectiveness in improving BMD and bone markers in HD and kidney transplant patients.

### Overall completeness and applicability of the evidence

The overall patients included in this review were recruited from seven different countries. Despite such variety, ethnicity was poorly reported, and most patients were male (range between 43 and 71%) The individual studies included in the current review are characterized by small sample sizes and mostly completed in HD patients, thus the summarized data may not apply to a broader adult population with CKD. Additionally, the methods used to assess PA and the exercise interventions varied substantially. Despite such lack of substantial studies, exercise is a non-pharmacological strategy widely recognized as a vital mechanical stimulus for the development and maintenance of optimal bone strength throughout life and the main results of this review point towards a positive effect of exercise on bone health in CKD patients, strengthening its usefulness and applicability within a clinical setting.

### Quality of the evidence

This systematic review included 13 studies, six observational (*n* = 367) and seven experimental (*n* = 215) studies. However, some of the included studies have methodological limitations that may limit their internal validity. PA was mainly assessed by self-report questionnaires and was poorly described. Regarding our main outcomes, most bone density data was measured with DXA scans, which do not distinguish between trabecular and cortical bone compartments and provide no measure of bone geometry. This may be interpreted as a limitation, as decreased bone strength in CKD patients is associated with the loss of both bone quantity (BMD) and quality (such as the bone microarchitecture) [10]. Furthermore, it has been proposed that DXA may erroneously attribute low BMD values in individuals with low volumetric density, due to a less accurate detection of bone edges, therefore underestimating BMD [33]. In addition, three studies had follow-up lengths of less than 16 weeks. As bone formation biologically takes around 4 months (approximately 16 weeks) to occur [34], interventions completed in shorter periods may not reliably detect skeletal changes with this imaging tool.

Additionally, the experimental studies scrutinised in this review examined the effects of different types of exercise, durations and intensities, and had an unclear risk of bias for most key domains. As an example, two of the experimental studies presented, despite a similar design (i.e.12-week, intradialytic exercise program), used different exercise modes (one aerobic [28], the other resistance [30]) and intensities, which may explain the different changes in BMD outcomes and the observed distinct risk of bias (unclear and high, respectively). Therefore, results should be interpreted with caution. Taken together, these methodological limitations impose some constraints to the quality of evidence summarized in the present review.

### Potential bias in the review process

A comprehensive search of journals in several databases was conducted and all published trials were identified. Different study designs were included in the review (i.e. longitudinal, cross-sectional, RCT and non-RCT), and all stages of the CKD as well as kidney transplant patients were considered. Additionally, the methodological quality of each study was corroborated by another reviewer. Some authors were contacted to clarify specific details required during data extraction and to ascertain if any newer data were available since publication. However, potential bias in this review exist. Unpublished trials were not included, as we did not conduct comprehensive searches of conference databases, and although we did not limit the searches to a particular language, only English language studies were included. We are unaware of any other potential biases in the review process.

### Agreements and disagreements with other studies or reviews

The link between PA and exercise with bone health has been extensively studied and summarized in several reviews [14, 15, 34, 35]. However, there are no previous reviews exploring this topic in CKD patients. Our systematic review demonstrated that only 3 studies in a total of 6 included in the review, suggests a positive association between PA and BMD at femoral neck and lumbar spine in HD [22, 25] and total body also in HD [25] and kidney transplant patients [20]. In fact, these findings are in agreement with other reviews in young adults [36] and post-menopausal women [37], suggesting that similar positive effects would also be expected in CKD patients. However, the lack of significant associations described in the current review, may be explained by the possible limited reliability of PA questionnaires to assess bone-specific loading exercise rather their intensity [38–40]. Previous studies support a relationship between PA and some bone markers such as BALP, OC in healthy subjects [41] and OPG in breast cancer [42] and post-menopausal [43] women, which is not corroborated by our present review in CKD patients.

Exercise trials in CKD patients were mostly based on resistance training protocols. This type of exercise has been shown to have a significant osteogenic effect [12]. Our review support that resistance exercise may be more effective in improving bone health outcomes than aerobic exercise, which is in agreement with the main literature in older adults and healthy adults [14, 44, 45]. Intradialytic resistance exercise training revealed to be effective in improving BMD at femoral neck and total body BMC in HD patients [30, 31], while resistance exercise performed by kidney transplant patients only improved proximal femur BMD [26]. Several systematic reviews in non-CKD subjects reported that high impact, resistance exercise or combined resistance with high impact exercise induced significant improvements in BMD at femoral neck of premenopausal women and older adults [13, 35, 45], which is in line with some of the results of this review.

A positive effect of resistance exercise on the regulation of the bone formation and resorption biomarkers in healthy subjects has been described [46]. Our findings also support that resistance exercise may elicit positive changes in bone metabolism in CKD patients, particularly in OPG [32], ALP [27] and BALP [29]. Of note, the impact of exercise in bone metabolism was not significant in all measured biomarkers (PTH, iPTH, TRAP5b, sclerostin, OC, OPN), which may be explained by differences in the detection of some biomarkers, measurement techniques, and exercise characteristics (type, intensity, duration and frequency).

## Conclusions

### Implications for practice

This is the first systematic review on observational and experimental studies to analyse the association of PA and exercise with bone outcomes and health in CKD patients. The main goal of the current review was to better inform about the association of PA with bone health and the exercise-related effects on bone health outcomes in CKD patients, and consequently to help improving exercise prescription recommendations.

Although the evidence summarized in this review on PA and bone health is limited, clinicians and exercise physiologists should advise CKD patients to increase their PA levels as it may be related with higher BMD, apart from other physiological and psychological benefits that may be derived from increasing PA.

Currently, CKD patients are advised to perform resistance exercise [47, 48] even though the exercise guidelines vary depending on the referenced organisation. However, most trials included in our review poorly described the resistance exercise protocols in terms of the exercise characteristics (i.e. mode, intensity, duration and frequency), and progression. The overall effects reported in this review pointed to an increase of BMD at femoral neck and proximal femur, which may prevent and/or decrease the risk of hip fractures. In addition, based on the current evidence, resistance exercise may increase OPG levels, which may be an indicator of better bone mass and strength. OPG protects bone from excessive resorption [49] and was recently associated with bone fractures in CKD patients [50]. Currently, there are no support for a positive effect of aerobic exercise in OPG levels [51].

This review included a limited number of resistance exercise trials; therefore, the summarized data is insufficient to support any recommendation on resistance exercise protocols as a more efficient intervention to improve bone health in CKD patients. Aerobic exercise is also broadly recommended for CKD patients [48], as it may be associated with better exercise capacity and physical functioning [52, 53]. Based on evidence from previous reviews in healthy adults, aerobic exercise of moderate intensity and low impact (such as walking) has limited effects on bone parameters [54–56]. Similar results should be expected in CKD patients.

### Implications for future research

This systematic review demonstrated that studies exploring this topic are currently lacking. Future studies should implement exercise interventions with a minimum duration of 16 weeks, using more sophisticated imaging techniques such as QCT or MRI, and including a set of key bone metabolism biomarkers of bone formation, resorption and CKD-MBD markers, as they quickly reveal changes in bone turnover. Useful bone biomarkers include: Type I Collagen Cross-Linked C-Telopeptide, P1NP ALP, BALP, OC, Trap-5b, Cathepsin K, sclerostin, DKK1, OPG, receptor activator of nuclear factor kappa-Β ligand (RANKL), RANKL/OPG ratio, among enzymes and nonenzymatic peptides derived from the cellular and noncellular compartments of bone.

Unfortunately, our summary of results showed inconsistent findings that were mostly based on small trials; thus, studies with appropriate statistical power are needed. In addition, most of the studies included in this review failed to describe the methods used to assess daily PA levels and the details of the exercise protocols and equipment. For instance, the use of elastic bands makes harder to define intensity and training progression. More RCTs exploring resistance exercise, aerobic, and combined exercise interventions exploring different intensities, durations, and in all spectrum of CKD disease, including pre-dialysis patients are clearly needed. Finally, the acute effects of exercise on bone health outcomes (mass quantity and quality, strength, and biomarkers) should be investigated.

## Supplementary information

**Additional file 1.** PRISMA Checklist. Twenty-seven-item checklist reporting of a systematic review.

**Additional file 2.** Search strategy. Report of a full electronic search strategy for EBSCO database.

**Additional file 3.** Adapted Newcastle-Ottawa Scale. Adapted scale to assess risk of bias of 7 observational studies included.

**Additional file 4.** Risk of bias summary of observational studies. Figure showing the summary of the risk of bias analysis observed in 7 observational studies.

**Additional file 5.** Risk of bias summary of experimental studies. Figure showing the summary of the risk of bias analysis observed in 6 experimental studies.

## Data Availability

The datasets generated during and/or analysed during the current study are available from the corresponding author on reasonable request.

## Abbreviations

ALP: Alkaline Phosphatase
BALP: Bone-specific Alkaline Phosphatase
BMC: Bone Mineral Content
BMD: Bone Mineral Density
BUA: Broadband Ultrasound Attenuation
CKD: Chronic Kidney Disease
DKK1: Dickkopf-related Protein 1
DXA: Dual X-ray absorptiometry
FGF-23: Fibroblast Growth Factor 23
HD: Haemodialysis
iP1NP: intact P1NP
iPTH: intact PTH
MBD: Mineral and Bone Disorders
MRI: Magnetic Resonance Imaging
Non-RCT: Non-randomized Control Trials
NOS: Modified Newcastle-Ottawa Scale
OC: Osteocalcin
OPG: Osteoprotegerin
OPN: Osteopontin
P1NP: Procollagen type I N-terminal Propeptide
PA: Physical Activity
PTH: Parathyroid Hormone
QCT: Quantitative Computerized Tomography
QUS: Quantitative Ultrasound
RANKL: Receptor Activator of Nuclear Factor Kappa-Β Ligand
RCTs: Randomized Control Trials
SOS: Speed of Sound
Trap-5b: Tartrate-resistant Acid Phosphatase Isoform 5b
