# Supplementary material for: Impact of physical activity and exercise on bone health in patients with chronic kidney disease: a systematic review of observational and experimental studies
Source: BMC Nephrol. 2020 Aug 8;21:334. doi: 10.1186/s12882-020-01999-z (PMC7414574; doi:10.1186/s12882-020-01999-z)
Supplement: Supplementary file 2 — Additional file 2. Search strategy. Report of a full electronic search strategy for EBSCO database. [file 12882_2020_1999_MOESM2_ESM.pdf]

Search Strategy: EBSCO

01: Exercise AND bone AND CKD
02: Exercise AND bone AND “renal function”
03: Exercise AND bone AND hemodialysis
04: Exercise AND bone AND dialysis
05: Exercise AND bone AND “glomerular filtration rate”
06: Exercise AND bone AND renal
07: “physical activity” AND bone AND CKD
08: “physical activity” AND bone AND “renal function”
09: “physical activity” AND bone AND hemodialysis
10: “physical activity” AND bone AND dialysis
11: “physical activity” AND bone AND “glomerular filtration rate”
12: “physical activity” AND bone AND renal
13: “physical performance AND bone AND CKD
14: “physical performance” AND bone AND “renal function”
15: “physical performance” AND bone AND hemodialysis
16: “physical performance” AND bone AND dialysis
17: “physical performance” AND bone AND “glomerular filtration rate”
18: “physical performance” AND bone AND renal
19: “physical function” AND bone AND CKD
20: “physical function” AND bone AND “renal function”
21: “physical function” AND bone AND hemodialysis
22: “physical function” AND bone AND dialysis
23: “physical function” AND bone AND “glomerular filtration rate”
24: “physical function” AND bone AND renal
25: “fitness” AND bone AND CKD
26: “fitness” AND bone AND “renal function”
27: “fitness” AND bone AND hemodialysis
28: “fitness” AND bone AND dialysis
29: “fitness” AND bone AND “glomerular filtration rate”
30: “fitness” AND bone AND renal
31: “functional ability” AND bone AND CKD
32: “functional ability” AND bone AND “renal function”
33: “functional ability” AND bone AND hemodialysis
34: “functional ability” AND bone AND dialysis
35: “functional ability” AND bone AND “glomerular filtration rate”
36: “functional ability” AND bone AND renal
