# Supplementary material for: Impact of physical activity and exercise on bone health in patients with chronic kidney disease: a systematic review of observational and experimental studies
Source: BMC Nephrol. 2020 Aug 8;21:334. doi: 10.1186/s12882-020-01999-z (PMC7414574; doi:10.1186/s12882-020-01999-z)
Supplement: Supplementary file 3 — Additional file 3. Adapted Newcastle-Ottawa Scale. Adapted scale to assess risk of bias of 7 observational studies included. [file 12882_2020_1999_MOESM3_ESM.pdf]

Tool for assessing risk of bias- Newcastle-Ottawa Scale modified

1- Selection Bias – Is the source population (cases, controls, cohorts) appropriate and representative of the population of interested? (Methods for selection study participants).

1 (low risk of bias) ☐

The investigators describe a:

- random selection from a population that is representative of the condition under study ☐
- A consecutive sample from a population that is representative of the condition under study ☐
- Eligible, exposed individuals from a defined population ☐

☐ Quote: " "

2 (moderate risk of bias) ☐

• A consecutive sample from a population that is not highly representative of the outcome of interest ☐

• A random selection from a population that is not highly representative of the outcome of interest ☐

☐ Quote: " "

3 (high risk of bias) ☐

Non-random approach, for example:

• The source population cannot be defined or enumerated ☐

• Volunteering or self-recruitment ☐

• No description of the derivation of the cohort ☐

☐ Quote: " "

? (unclear risk of bias) ☐

• Insufficient information to permit judgment of '1' , '2' or '3' ☐

2- **Performance Bias** (Methods to control for confounding)

2.1. Is the sample size sufficient and is there sufficient power to detect a meaningful difference in the outcome of interest?

1 (low risk of bias) ☐

• Sample size was adequate and there was sufficient power to detect a difference in the outcome ☐<br>☐ Quote: " "

2 (moderate risk of bias) ☐

• Sample size may not be adequate and study may have been slightly underpowered ☐<br>☐ Quote: " "

3 (high risk of bias) ☐

• Sample size was small and there was not enough power to test the outcome of interest ☐<br>☐ Quote: " "

? (unclear risk of bias) ☐

• Insufficient information to permit judgment of '1' , '2' or '3' ☐

2.2. Did the study adjust for any variables or confounders that may influence the outcome?

1 (low risk of bias) ☐

• The study identified and adjusted for all possible confounders that may influence the estimates of association between exposure and outcome ☐<br>☐ Quote: " "

2 (moderate risk of bias) ☐

• The study identified and reported possible variables that may influence the outcome but did not statistically explore their influence☐<br>☐ Quote: " "

3 (high risk of bias) ☐

• The study either did not report any variables of influence or acknowledge any variables of influence when it was clear they were present.☐

☐ Quote: " "

? (unclear risk of bias) ☐

• Insufficient information to permit judgment of '1', '2' or '3' ☐

3- **Detection bias** (Statistical methods)

3.1. Did the study use appropriate statistical analysis methods relative to the outcome of interest?

1 (low risk of bias) ☐

Any one of the following:

• The study reported use of appropriate statistical analysis as required ☐

☐ Quote: " "

2 (moderate risk of bias) ☐

• The study used either correct statistical methods but did not report them well, or used the incorrect methods but reported them in detail ☐

☐ Quote: " "

3 (high risk of bias) ☐

• The study did not use appropriate statistical analysis as required ☐

☐ Quote: " "

? (unclear risk of bias) ☐

• Insufficient information to permit judgment of '1', '2' or '3' ☐

3.2. Is there little missing data and did the study handle it accordingly?

1 (low risk of bias) ☐

• The study acknowledged missing data to be less than 10% and specified the method of handling it ☐

• No missing data ☐

☐ Quote: " "

2 (moderate risk of bias) ☐

• The study either had greater than 15% of missing data but they specified the method used to handle it ☐<br>• Missing data is not excessive, and they specified the method used to handle it ☐<br>☐ Quote: " "

3 (high risk of bias) ☐

• The study had greater than 15% of missing data and did not handle it at all ☐<br>☐ Quote: " "

? (unclear risk of bias) ☐

• Insufficient information to permit judgment of '1' , '2' or '3' ☐

4- Information bias (Methods of measuring outcome variables)

4.1. Is the methodology of the outcome measurement explicitly stated and is it appropriate?

1 (low risk of bias) ☐

• The study provides a detailed description of the outcome measure(s) which are appropriate for the outcome of interest ☐<br>☐ Quote: " "

2 (moderate risk of bias) ☐

• The study provides a somewhat complete description of outcome measurements that are justified<br>☐<br>☐ Quote: " "

3 (high risk of bias) ☐

• The study provides limited information on the methods of measuring the outcome and the measure is not appropriate considering the outcome ☐<br>☐ Quote: " "

? (unclear risk of bias) ☐

• Insufficient information to permit judgment of '1' , '2' or '3' ☐

4.2. Is there an objective assessment of the outcome of interest?

1 (low risk of bias) ☐

• The study used objective methods to discern the outcome status of participants (i.e. laboratory measurements, medical records) ☐<br>☐ Quote: " "

2 (moderate risk of bias) ☐

• The study relied on subjective data as the primary method to discern the outcome status of participants (i.e. self-report) ☐<br>☐ Quote: " "

3 (high risk of bias) ☐

• The study had limited reporting about assessment of outcomes ☐<br>• No description ☐<br>☐ Quote: " "

? (unclear risk of bias) ☐

• Insufficient information to permit judgment of '1' , '2' or '3' ☐

5- Attrition bias (Subject follow-up)

5.1. Was the follow-up sufficiently long enough for the outcome to occur?

1 (low risk of bias) ☐

• Follow-up was sufficiently long enough for the outcome to occur ☐<br>☐ Quote: " "

3 (high risk of bias) ☐

• Follow-up was not sufficiently long enough for the outcome to occur ☐<br>☐ Quote: " "

? (unclear risk of bias) ☐

• Insufficient information to permit judgment of '1' or '3' ☐

5.2. Was there minimal loss to follow-up and are subjects lost to follow-up unlikely to introduce bias?

1 (low risk of bias) ☐

• Follow-up was completed for all, or nearly all subjects, and reasons for losses to follow-up were well documented. ☐<br>☐ Quote: " "

2 (moderate risk of bias) ☐

• Losses to follow-up are not excessive, and reasons for losses to follow-up are well documented and mostly unrelated to the outcome ☐<br>☐ Quote: " "

3 (high risk of bias) ☐

• Significant loss to follow-up, reasons for losses to follow-up not reported, suspect that reasons for dropouts are related to the outcome ☐<br>☐ Quote: " "

? (unclear risk of bias) ☐

• Insufficient information to permit judgment of '1', '2' or '3' ☐
