# Supplementary figures and images for: Impact of physical activity and exercise on bone health in patients with chronic kidney disease: a systematic review of observational and experimental studies
Source: BMC Nephrol. 2020 Aug 8;21:334. doi: 10.1186/s12882-020-01999-z (PMC7414574; doi:10.1186/s12882-020-01999-z)

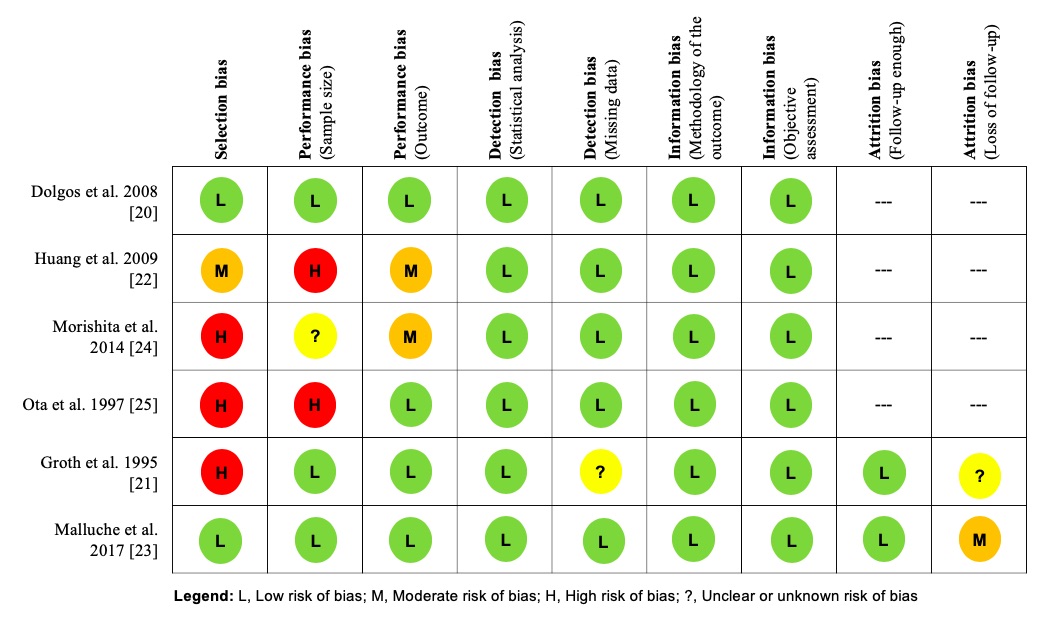

Supplement: Supplementary file 4 — Additional file 4. Risk of bias summary of observational studies. Figure showing the summary of the risk of bias analysis observed in 7 observational studies. [file 12882_2020_1999_MOESM4_ESM.jpg]

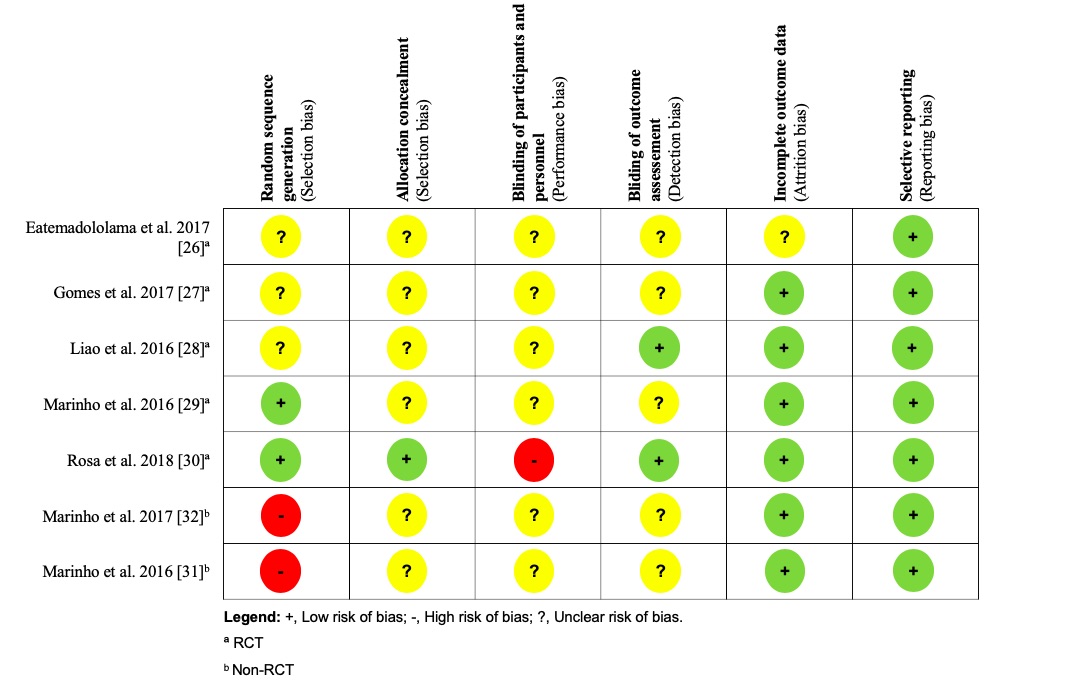

Supplement: Supplementary file 5 — Additional file 5. Risk of bias summary of experimental studies. Figure showing the summary of the risk of bias analysis observed in 6 experimental studies. [file 12882_2020_1999_MOESM5_ESM.jpg]
